# Diphenyleneiodonium Triggers Cell Death of Acute Myeloid Leukemia Cells by Blocking the Mitochondrial Respiratory Chain, and Synergizes with Cytarabine

**DOI:** 10.3390/cancers14102485

**Published:** 2022-05-18

**Authors:** Hassan Dakik, Maya El Dor, Jérôme Bourgeais, Farah Kouzi, Olivier Herault, Fabrice Gouilleux, Kazem Zibara, Frédéric Mazurier

**Affiliations:** 1 EA7501 GICC/CNRS ERL7001 LNOx, University of Tours, F-37032 Tours, France; hassan.dakik@mail.mcgill.ca (H.D.); maya.el-dor@etu.univ-tours.fr (M.E.D.); j.bourgeais@chu-tours.fr (J.B.); farah.kouzi@etu.univ-tours.fr (F.K.); olivier.herault@univ-tours.fr (O.H.); fabrice.gouilleux@univ-tours.fr (F.G.); 2 Department of Biological Hematology, Tours University Hospital, F-37000 Tours, France; 3 Biology Department, Faculty of Sciences, Lebanese University, Beirut 90656, Lebanon; 4 ER045, PRASE, Beirut 6573/14, Lebanon

**Keywords:** DPI, mitochondria, leukemia, oxidative stress, OxPhos, Ara-C

## Abstract

**Simple Summary:**

Acute myeloid leukemia (AML) is an aggressive heterogeneous cancer of the blood, of which 70% of cases develop relapse. Relapse is mainly due to chemoresistant leukemic cells (LCs) that are characterized by high mitochondrial oxidative phosphorylation (OxPhos) status, i.e., cells that are dependent on the mitochondrial respiratory chain (MRC) function. The aim of our study was to determine whether diphenyleneiodonium (DPI)—known as a potent inhibitor of flavoproteins—could be used to target AML cells. Herein, we demonstrate that DPI disrupts the mitochondrial function of AML cell lines. Interestingly, we found that cells with high OxPhos are more sensitive to the apoptotic effects of DPI. Moreover, we showed that DPI sensitizes AML cell lines to cytarabine (Ara-C) treatment, suggesting that MRC inhibitors could be employed to target LCs that are resistant to this chemotherapeutic agent.

**Abstract:**

Acute myeloid leukemia (AML) is characterized by the accumulation of undifferentiated blast cells in the bone marrow and blood. In most cases of AML, relapse frequently occurs due to resistance to chemotherapy. Compelling research results indicate that drug resistance in cancer cells is highly dependent on the intracellular levels of reactive oxygen species (ROS). Modulating ROS levels is therefore a valuable strategy to overcome the chemotherapy resistance of leukemic cells. In this study, we evaluated the efficiency of diphenyleneiodonium (DPI)—a well-known inhibitor of ROS production—in targeting AML cells. Results showed that although inhibiting cytoplasmic ROS production, DPI also triggered an increase in the mitochondrial ROS levels, caused by the disruption of the mitochondrial respiratory chain. We also demonstrated that DPI blocks mitochondrial oxidative phosphorylation (OxPhos) in a dose-dependent manner, and that AML cells with high OxPhos status are highly sensitive to treatment with DPI, which synergizes with the chemotherapeutic agent cytarabine (Ara-C). Thus, our results suggest that targeting mitochondrial function with DPI might be exploited to target AML cells with high OxPhos status.

## 1. Introduction

AML is a heterogeneous clonal disorder of myeloid progenitors that accumulate due to a blockage in their differentiation, leading to death [1]. AML therapy has not changed much over the last several decades, and more than 70% of AML patients relapse within 3 years after therapy [2]. AML relapse is caused by residual populations of quiescent leukemic stem cells (LSCs), associated with chemoresistant AML cells that have high mitochondrial oxidative phosphorylation [3,4]. Altered cellular redox status with high ROS levels is indeed a common hallmark of AML cells. Several lines of evidence have indicated that NADPH oxidase (NOX) complexes that are major contributors to the production of ROS—including superoxide (O_2_^•−^) and hydrogen peroxide (H_2_O_2_)—are also important regulators of AML progression and drug resistance [5,6]. Thus, targeting oxidative metabolism in AML has been proposed as a promising therapeutic strategy to eradicate AML cells [7].

We recently showed that all components of NOX2—the most prominent NOX complex across AML—are highly expressed at both the transcriptional and protein levels. Surprisingly, we did not find detectable constitutive NOX activity in 24 leukemic cell lines [8]. In addition, we demonstrated that NOX2 silencing neither affected AML cell growth nor triggered cell death in vitro. Adane et al. have recently shown that, although silencing of NOX2 induces the differentiation of primary AML cells, DPI used to inhibit NOX did not affect the differentiation, but triggered apoptosis [9]. While DPI is widely used to prove NOX activity [10], it is a non-specific inhibitor of flavoproteins that can impede the activity of nitric oxide synthases (NOS), xanthine oxidases (XOS), and complexes I and III of the mitochondrial respiratory chain (MRC) [11,12,13]. Moreover, it can form phenol radicals, thus promoting off-target effects, including reduced flavin (FAD or FMN) of NOX or P-450 reductase [14,15], the heme component of NOX [16], or iron–sulfur clusters in mitochondrial complex I. Importantly, DPI has been found to trigger the inhibition of the mitochondrial OxPhos in breast cancer cells, and to induce a chemo-quiescent phenotype that blocks the propagation of cancer stem cells [17].

Recently, it has been proposed that drug resistance of AML cells might be dependent on their OxPhos status [3]. Hence, we hypothesized that DPI could target the OxPhos system in AML cells independently of its capacity to inhibit the NOX complexes. Thus, we examined the effects of DPI on oxidative metabolism, proliferation, and resistance to chemotherapy in various AML cell lines harboring low- and high-OxPhos phenotypes.

## 2. Results

### 2.1. DPI Reduces Cytoplasmic ROS while Inducing Superoxide Production

**Figure 1:**
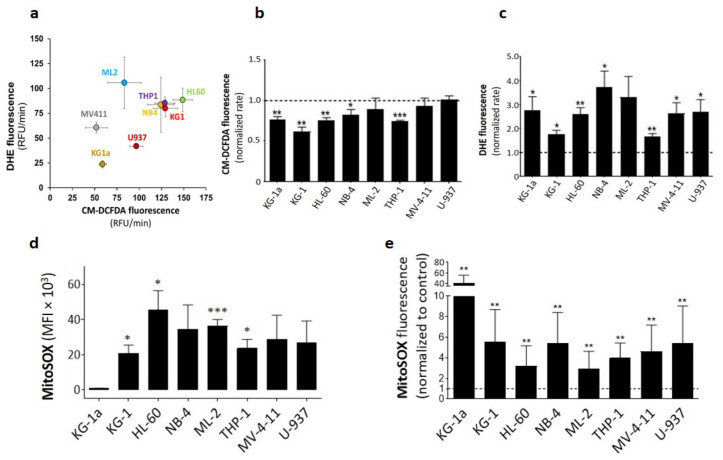
Steady-state and DPI-induced ROS production in eight AML cell lines: (**a**) ROS production rates presented as DHE vs. H_2_CM-DCFDA fluorescence rates. ROS production rates (RFU/min) were calculated from the curve’s slope over 1 h using CLARIOstar data analysis software. (**b**) Effect of DPI (20 μM) on ROS production rates measured by CM-DCFDA fluorescence (*n* = 3). (**c**) Effect of DPI (20 μM) on ROS production rates measured by DHE fluorescence (*n* = 3). DPI data are shown as normalized fluorescence rates with respect to the control for each cell line. (**d**) Baseline mitochondrial ROS production measured by MitoSOX fluorescence (**d**). Student’s *t*-test was used to compare MitoSOX levels in various cell lines to those of KG-1a cells (*n* = 4). (**e**) Effect of DPI on mitochondrial ROS production (*n* = 4). Data are shown as mean values ± SEM. In panels (**b**,**c**,**e**), a one-sample *t*-test was used to compare normalized rates to 1 (* *p* < 0.05; ** *p* < 0.01; *** *p* < 0.001).

To study the effect of DPI on AML cells, we used eight AML cell lines, covering FAB stages M0–M5 [18], and with no endogenous NOX activity [8]. First, we measured ROS production rates using CM-H_2_DCFDA, which detects cytoplasmic ROS (cytoROS) production—mainly H_2_O_2_—and dihydroethidium (DHE), which detects intracellular superoxide (O_2_^•−^) (Figure S1). We noticed high heterogeneity in ROS production between the cell lines (Figure 1a). KG-1, HL-60, NB-4, and THP-1 cells clustered together, showing concomitant high production rates of O_2_^•−^ and cytoROS, suggesting steady transformation of O_2_^•−^ into H_2_O_2_. Markedly, KG-1a cells (M0)—a model of immature AML, derived from KG-1—showed low production rates of superoxide (23 RFU/min) and cytoROS (59 RFU/min). This is consistent with the idea that more mature cells have higher ROS levels [5,6,19]. After treatment with 20 µM DPI—a dose sufficient to inhibit flavoproteins—all cell lines showed a substantial increase in O_2_^•−^ production, while only five of them had decreased cytoROS production rates, compared to their respective controls (Figure 1b,c). To investigate the origin of the O_2_^•−^ increase following DPI treatment, we measured mitochondrial O_2_^•−^ (mitoROS) production using MitoSOX—a DHE derivative that is specific to mitochondria. At a steady state, the profile of O_2_^•−^ production detected by MitoSOX was concordant with that obtained by DHE (Figure 1d). Interestingly, KG-1a cells had the lowest levels. Remarkably, DPI treatment triggered a strong induction of mitoROS levels in all cell lines (Figure 1e). Although KG-1a cells showed the strongest induction (40-fold) with DPI, their increased mitoROS levels never reached the baseline levels of the other cell lines. Together, these data show that DPI decreases cytoplasmic ROS production but concomitantly triggers an increase in mitochondrial ROS production.

### 2.2. DPI Disrupts the Mitochondrial Membrane Potential

**Figure 2:**
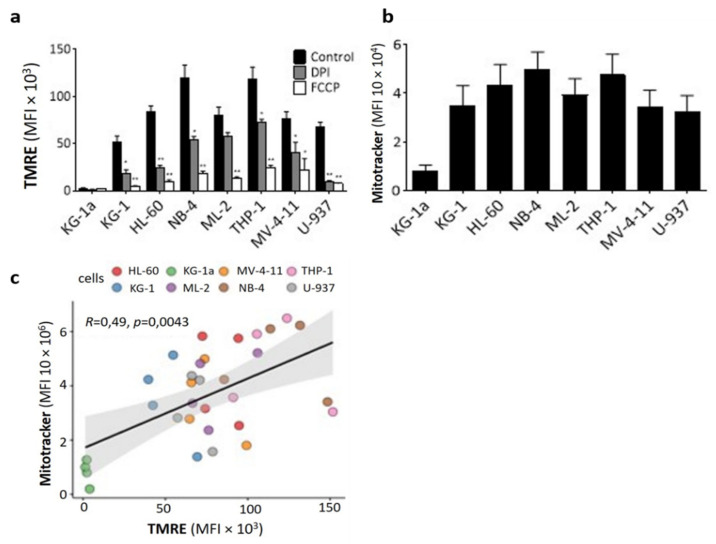
Effect of DPI on mitochondrial membrane potential (ΔΨm): (**a**) Effect of DPI on ΔΨm as measured by TMRE fluorescence. FCCP was used as a positive control (20 uM). (**b**) Physiological mitochondrial mass in the indicated AML cell lines, as measured by MitoTracker fluorescence. (**c**) Spearman’s correlation analysis of ΔΨm and mitochondrial mass, as measured by TMRE and MitoTracker, respectively, determined in a and b. Dots represent cells from independent experiments. Data are shown as mean values ± SEM (*n* = 4). Student’s *t*-test was used to compare treated conditions to controls (* *p* < 0.05; ** *p* < 0.01).

To explain the quick mitoROS burst induced by DPI, we speculated that DPI may have induced oxidative stress by disrupting the MRC. We thus examined the functional impact of DPI on the mitochondrial activity of AML cells by labelling with tetramethylrhodamine ethyl ester (TMRE) as a readout to determine the effects on mitochondrial membrane potential (ΔΨm). FCCP, a common mitochondria-depolarizing agent, was used as a positive control. The basal level of ΔΨm was variable across the cell lines (Figure 2a; control black bars). This variability was mainly due to differences in the mitochondrial mass, as determined by MitoTracker Deep Red labelling (Figure 2b,c). DPI notably decreased ΔΨm in all cell lines, except in KG-1a cells, in which ΔΨm was negligible, in accordance with the lowest mitochondrial biomass being found in this cell line (Figure 2a–c). Collectively, these data indicate that DPI triggers O_2_^•−^ production in AML cell lines by inhibiting the MRC.

### 2.3. DPI Disrupts the MRC and Alters the Energetic Metabolism

**Figure 3:**
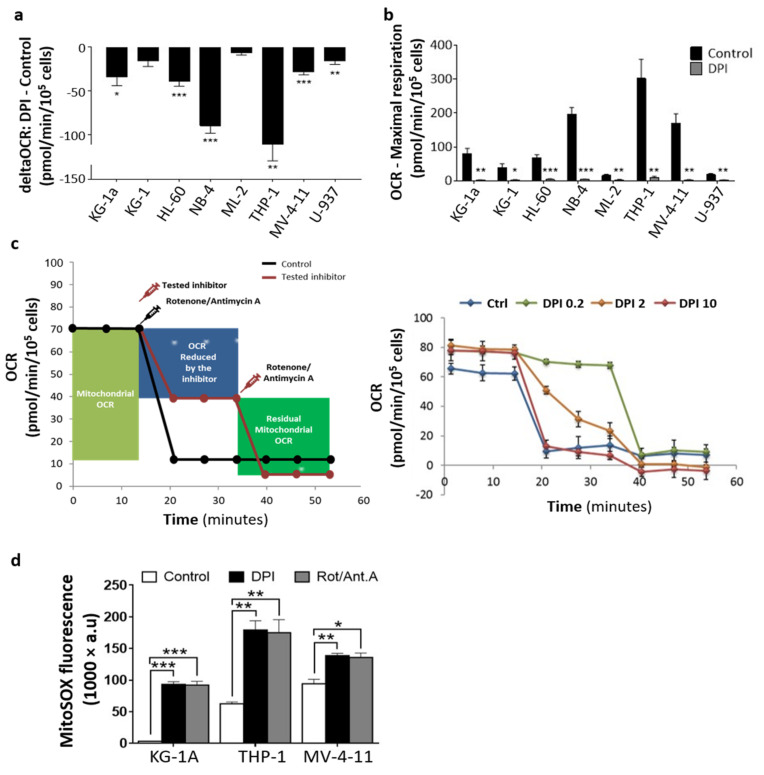
Effect of DPI on mitochondrial oxygen consumption rate (OCR): (**a**) Quantification of the acute effect of DPI on the OCR of eight AML cell lines. (**b**) Quantification of the effect of DPI on the maximal respiration of the same cell lines. (**c**) Left: schematic representation of the experimental design to measure the OCR IC50 values for DPI, rotenone, and antimycin A. Right: a representative experiment showing the OCR kinetic response of the THP-1 cell line to selected DPI doses (0.2, 2, and 10 μM). Mitochondrial OCR was calculated for each cell line from the control condition after injection of a mixture of rotenone (0.5 μM) and antimycin A (0.5 μM). Residual mitochondrial OCR was deduced from the inhibitor condition after a secondary injection of a mix of rotenone (0.5 μM) and antimycin A (0.5 μM). (**d**) Effect of DPI (20 μM) on mitoROS in the three cell lines in comparison with rotenone (0.5 μM)/antimycin A (0.5 μM) combination. Data are shown as mean values ± SEM (*n* = 3). In (**a**), a one-sample *t*-test was used to compare the delta OCR values to 0. In (**b**,**d**), Student’s *t*-test was used to compare treated conditions to controls (* *p* < 0.05; ** *p* < 0.01; *** *p* < 0.001).

To gain insights into the effect of DPI on MRC, we performed bioenergetic analyses of OxPhos through the measurement of oxygen consumption rate (OCR), using Seahorse technology. DPI significantly reduced the OCR of six out of the eight cell lines (Figure 3a), and reduced the maximal respiration capacity in all cell lines (Figure 3b). To examine the OCR inhibition efficiency of DPI, we calculated its IC50 values alongside the standard inhibitors antimycin A and rotenone in three representative cell lines (Figure 3c; THP-1 and MV-4-11 with high OCR, and KG-1a with low OCR). The results showed that DPI had IC50 values for OCR (0.2–1.29 µM) in the same range as those of antimycin A (0.26–0.53 µM) and rotenone (0.55–1.22 µM), indicating that DPI is as efficient as current respiratory chain inhibitors (Table 1). Finally, to prove that blocking OCR may induce mitoROS, the three cell lines were treated with either DPI or a combination of rotenone and antimycin. As expected, all inhibitors triggered a similar increase in mitoROS levels (Figure 3d). Together, these data indicate that DPI blocks the MRC in AML cell lines, and induces an oxidative burst with a similar efficiency to standard inhibitors.

### 2.4. DPI Reduces Cell Proliferation and Triggers Apoptosis

**Figure 4:**
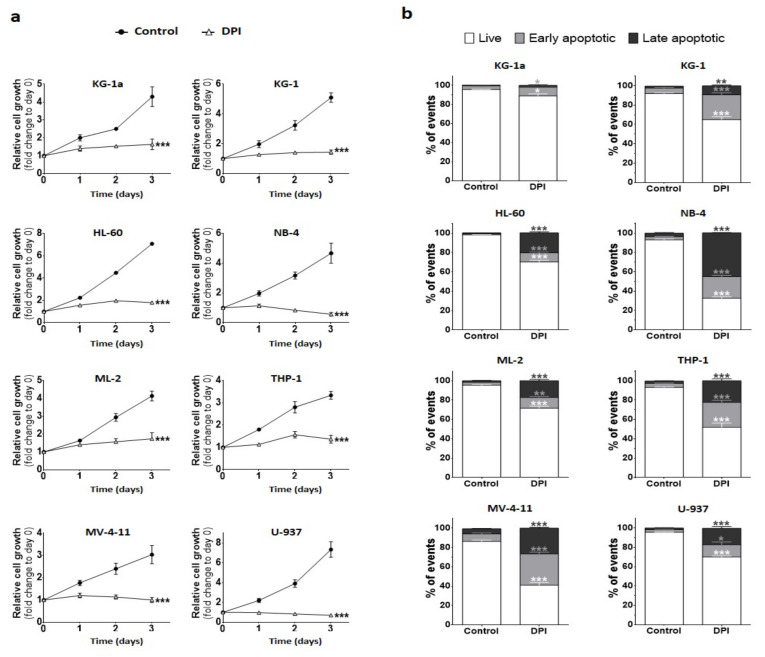
Effect of DPI on cell proliferation and apoptosis: Cell growth was assessed using resazurin reduction assay at the indicated days, following DPI (0.4 μM) treatment. Analysis of apoptosis was performed at day 3 after treatment. (**a**) Growth curves from various conditions for eight AML cell lines. Relative cell growth was calculated as resazurin fluorescence fold change compared to the control at day 0. Data are shown as means ± SEM (*n* = 3). Two-way ANOVA was performed for each cell line, followed by Tukey’s post hoc analysis. Adjusted *p*-values are shown from day 3 comparing DPI condition to the DMSO control (* *p* < 0.05; ** *p* < 0.01; ***, *p* < 0.001). (**b**) Apoptosis in DPI-treated AML cell lines. 7-AAD/Annexin-V staining distinguishes between live, early-apoptotic, and late-apoptotic cells. Data are shown as means ± SEM (*n* = 3 independent experiments). Student’s *t*-test was used to compare DPI conditions to their corresponding control counterparts (* *p* < 0.05; ** *p* < 0.01; *** *p* < 0.001).

Since DPI induced oxidative stress by blocking mitochondrial respiration, we examined its impact on cell growth and survival. To address the effect of chronic exposure, we used a low dose of DPI (0.4 μM), and observed the proliferation of AML cell lines for three days (Figure S2). The results showed that all cell lines exposed to DPI had a significant reduction in their expansion capacity compared to their corresponding controls (Figure 4a). Furthermore, to investigate whether reduced expansion resulted from proliferation slowdown or the induction of cell death, we quantified apoptosis at day 3 of culture, using Annexin-V and 7-AAD (Figure 4b). Following DPI treatment, most cell lines showed moderate-to-high levels of apoptosis that could partially explain the reduction in cell growth (Figure 4a,b). NB-4, THP-1, and MV-4-11 cells, which showed the highest apoptosis rates (Figure 4b), were also those with the highest maximal OCR capacities (Figure 3b). In contrast, KG-1a cells that showed low mitoROS and minimal ΔΨm (Figure 1d and Figure 2a) also showed minimal apoptosis (Figure 4b). Together, these data suggest that DPI reduces cell growth by inhibiting cell division in an apoptosis-dependent manner, and that cells with high OxPhos metabolism are more sensitive to DPI-induced apoptosis.

### 2.5. Effects of Combination Therapy of DPI and Cytarabine on AML Cell Lines

**Figure 5:**
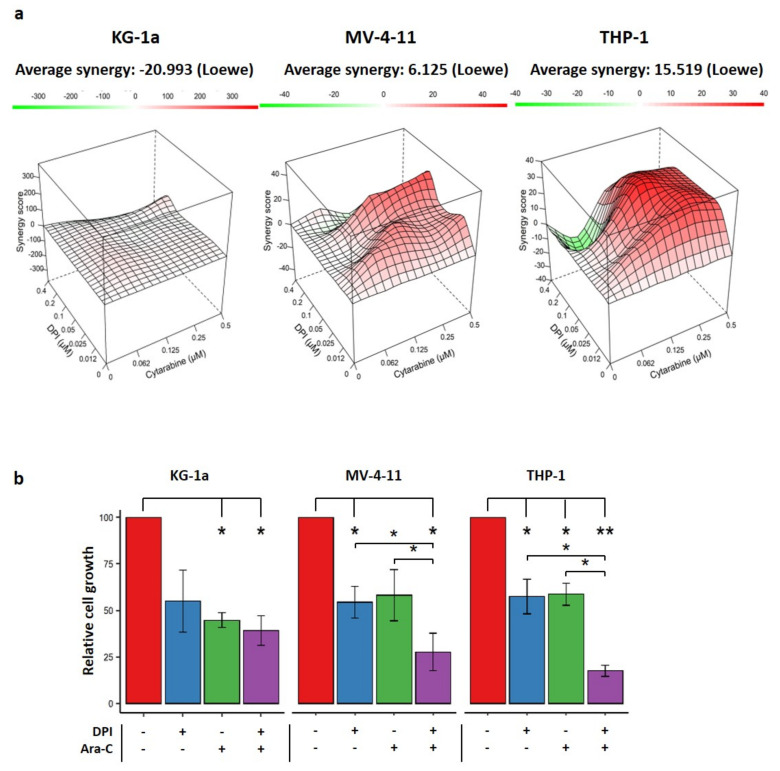
Effect of combination therapy of DPI and Ara-C: (**a**) 3D interaction landscape showing Loewe’s synergy score for the effect of the combination of various doses of DPI and Ara-C on growth inhibition of KG-1a, THP-1, and MV-4-11 cell lines. The synergy score on the Z-axis corresponds to excess % inhibition beyond the expectation set by the Loewe additivity equation. This score reflects the percentage of growth inhibition that can be attributed to the drug interactions. An average synergy score is shown over the dose–response matrix. Data are shown as the average from 3 independent experiments. (**b**) Bar plots showing the effects of DPI (0.1 µM), Ara-C (0.25 µM), or their combination on cell growth, as compared to controls. Data are shown as mean values ± SEM (*n* = 3). Student’s *t*-test followed by BH adjustment was used for pairwise comparisons (* *p* < 0.05; ** *p* < 0.01).

Recent findings have suggested that AML cells with high OxPhos are more resistant to therapeutic agents [3,4]. Therefore, we investigated whether DPI could synergize with Ara-C to eliminate AML cells. To address this issue, we used two representative cell lines (THP-1 and MV-4-11) with high OxPhos, and KG-1a, with the lowest OxPhos status. A dose–response matrix was designed to test 35 different combinations of doses, ranging from 0 to 0.5 µM for Ara-C and from 0 to 0.4 µM for DPI (Figure 5a). Data showed that the combination of DPI and Ara-C resulted in a synergistic effect on THP-1 and MV-4-11 cells (positive Loewe scores), but not on KG-1a cells (a negative score) (Figure 5a,b). This suggests that only the cell lines with high OCR could be sensitized by DPI.

## 3. Discussion

Previous studies have shown that DPI reduces leukemic cell viability, suggesting that NOX2 could contribute to primary human leukemia cells’ survival and proliferation [9]. Moreover, treatment of K562 cell xenograft models in CB17-SCID mice with DPI significantly slowed tumor growth, thus suggesting NOX inhibitors as a good strategy for CML treatment [20]. However, DPI has demonstrated its potential to block the function of MRC complex I in mitochondria isolated from rat skeletal muscles [13]. It has been suggested that DPI acts by inhibiting flavin-containing enzymes, which are dependent on FMN and FAD [21]. Therefore, we hypothesized that the previously described anti-leukemic effect of DPI could have been independent of anti-NOX activity, and we sought to understand its mechanism of action in AML cell lines with no detectable NOX activity. We demonstrated that DPI, although inhibiting cytoplasmic ROS production, disrupts the MRC, increases mitochondrial ROS production, and triggers the apoptosis of AML cell lines—especially those with a high OxPhos status. In addition, we showed that DPI synergizes with Ara-C in preferentially eliminating high-OxPhos AML cells.

First, we showed that DPI reduces cytoROS production in five of the eight tested cell lines. This decrease was previously shown in three leukemic cell lines (KU-812, MOLM-13, and HEL) treated with DPI [22]. DPI has also been found to reduce the cytoROS production, thereby impacting the proliferation of prostate cancer cells [23]. Although an increase in mitochondrial superoxide would be rationally expected to accompany a concomitant increase in cytoplasmic H_2_O_2_, our results showed that DPI gives rise to an increase in mitoROS, can inhibit the mitochondrial respiration, and induces apoptosis. This is consistent with the data showing that DPI induces cell cycle arrest and decreases the mitochondrial membrane potential of prostate cancer cell lines [24]. Redox homeostasis is a complex mechanism governed by the finely tuned balance between pro-oxidant and antioxidant systems. The lack of increase in cytoROS levels might indicate limited transformation of superoxide into H_2_O_2_ by the mitochondrial superoxide dismutase (SOD2), or an efficient elimination of generated H_2_O_2_ by catalase in the cytoplasm. We have recently shown that AML cell lines express high levels of antioxidant enzymes, including SOD2 and catalase [25]. This indicates that low cytoplasmic ROS levels could be driven by efficient elimination rather than by the lack of transformation into H_2_O_2_. Alternatively, DPI could have inhibited other flavoproteins in the cytoplasm (such as XDH or NOS) that are also known to produce ROS. Ozsvari et al. also demonstrated that the treatment of breast cancer cell lines with DPI inhibits mitochondrial oxidative metabolism (OxPhos), thereby reducing mitochondrial ATP production by more than 90% [17]. However, DPI did not trigger the production of mitochondrial ROS in these cells, which could be explained by the use of a very low dose (10 nM). Collectively, these data show that DPI can efficiently inhibit mitochondrial respiration in different types of cancer cells, even at very low doses.

DPI readily impeded the mitochondrial respiration in high-OxPhos AML cells, independently of NOX inhibition, causing a strong burst of superoxide production. We previously reported that another NOX inhibitor, VAS3947, induces the apoptosis of AML cells through cysteine thiol alkylation, independently of NOX inhibition [25]. Several studies have indicated that NOX complexes are important regulators of AML progression and drug resistance [9,19,20,26,27,28,29], but many of these used non-specific inhibitors—including DPI and VAS3947—to prove NOX activity or to study the functional impact of NOX inhibition on leukemic cells. Our findings suggest that the use of such inhibitors to study the role of NOX in oxidative metabolism can be misleading, and highlight the need to develop more specific NOX inhibitors or to use knockdown strategies.

To examine the synergy between DPI and cytarabine, we implemented the Loewe additivity method [30]. According to this method, the combination of DPI and Ara-C is synergistic in THP-1 cells, and even in MV-4-11 cells—with both having a high OxPhos metabolism—but not in low-OxPhos cells (e.g., KG-1a). The combined treatment of DPI with FLT3-ITD inhibitors, such as midostaurin or sorafenib, has also been proposed to synergistically inhibit the proliferation of AML cell lines harboring FLT3-ITD, and its combination with the tyrosine kinase inhibitor imatinib has been shown to synergistically increase apoptosis in chronic myeloid leukemia (CML) cells in vivo [20]. Remarkably, the authors have demonstrated that the viability of healthy CD34-positive cells is not affected by DPI, suggesting that this compound might be safely used in the treatment of myeloid leukemia. Recent reports have revealed that chemoresistance and relapse may arise from cells bearing a high OxPhos metabolism [3,4]. Notably, Ara-C-resistant AML populations exhibit metabolic characteristics and gene signatures compatible with a high OxPhos status [3]. In these cells, targeting the mitochondrial metabolism induced an energetic shift towards low OxPhos, and enhanced the anti-leukemic effects of Ara-C [3]. Altogether, these data support our findings that targeting the high OxPhos status of AML cells might help to overcome their resistance to chemotherapy.

In summary, this work reports that DPI’s anti-leukemic activity is caused by the inhibition of the MRC, along with OxPhos disruption. We also found that DPI can synergize with Ara-C in targeting high-OxPhos AML cells. Thus, our data pave the way for the future development of therapies that specifically target mitochondrial respiration in myeloid leukemias.

## 4. Materials and Methods

### 4.1. Cell Lines and Culture

Human myeloid leukemia cell lines (KG-1a, KG-1, HL-60, NB-4, ML-2, THP-1, MV-4-11, and U-937) were purchased from DSMZ (German Collection of Microorganisms and Cell Cultures, Braunschweig, Germany). Cells were cultured in RPMI medium (Life Technologies, Villebon-sur-Yvette, France) supplemented with 10% heat-inactivated fetal bovine serum (FBS, Life Technologies), 2 mM L-glutamine (Life Technologies), 100 U/mL penicillin (Sigma-Aldrich, Saint-Quentin-Fallavier, France), and 100 µg/mL streptomycin (Boehringer-Mannheim, Meylan, France) at 37 °C in fully humidified air and 5% CO_2_. For all experiments, cells were harvested from culture while in their exponential growth phase.

### 4.2. ROS Measurement

#### 4.2.1. Fluorometric Assay (CM-DCFDA and DHE)

Cells were washed with PBS and seeded in a 96-well plate at 2.10^5^ cells per well in a 200 μL reaction volume, in the presence or absence of DPI (20 μM) (Sigma-Aldrich, Saint-Quentin-Fallavier, France). DMSO was used as a vehicle control. Cytoplasmic ROS and intracellular superoxide production was detected with 5-(and-6)-chloromethyl-2′,7′-dichlorodihydrofluorescein diacetate (CM-DCFDA) (Life Technologies) and dihydroethidium (DHE) (Invitrogen, Villebon sur Yvette, France), respectively, each at a concentration of 5 μM per well. ROS production was measured kinetically for CM-DCFDA (λex = 483-14 nm, λem = 530-30 nm) and DHE (λ_ex_ = 490-15 nm, λ_em_ = 600-20 nm) every minute for 1 h at 37 °C using a CLARIOstar plate reader (BMG Labtech, Champigny-sur-Marne, France). ROS production rates (RFU/min) were calculated via the CLARIOstar data analysis software, using the slope of the curve at steady state.

#### 4.2.2. Flow Cytometry Assay (MitoSOX)

Cells were washed with PBS and resuspended at 1.10^5^ cells in a 100 μL reaction volume, in the presence or absence of DPI (20 μM). DMSO was used as a vehicle control. Mitochondrial superoxide production was measured using MitoSOX (Life Technologies), which was added at 5 μM per condition. The samples were incubated in the dark for 30 min at 37 °C prior to fluorescence measurement with a C6 Accuri^®^ flow cytometer (Becton Dickinson, Le Pont de Claix, France). Data were analyzed using FlowJo^®^ software v.10 (Becton Dickinson).

### 4.3. Mitochondrial Function Measurement

#### 4.3.1. Mitochondrial Mass

The relative quantification of mitochondrial mass was evaluated using the fluorescent probe MitoTracker Deep Red (Invitrogen), according to the supplier’s instructions. Briefly, cells (1.10^5^) were harvested from culture and then stained with MitoTracker (50 nM) in 200 μL of fresh RPMI medium without FBS for 30 min at 37 °C in the dark. Cells were then washed in warm PBS, and their fluorescence intensity was measured by flow cytometry with a C6 Accuri^®^ flow cytometer. DMSO was used as a vehicle control. FlowJo^®^ software was used for data analysis.

#### 4.3.2. Mitochondrial Membrane Potential

Mitochondrial membrane potential (ΔΨm) was assessed using the fluorescent probe tetramethylrhodamine ethyl ester (TMRE; Invitrogen). A total of 1.10^5^ cells was resuspended in fresh RPMI medium supplemented with 10% FBS and stained with TMRE (100 nM), in the presence or absence of DPI (20 μM), at a volume of 200 μL for 30 min at 37 °C in the dark. Cells were then washed in warm PBS, and their fluorescence intensity was measured by flow cytometry with a C6 Accuri^®^ flow cytometer. FCCP (20 µM) (Selleckchem, Houston, TX, USA) was used as a positive control and DMSO was used as a vehicle control. FlowJo^®^ software was used for data analysis.

### 4.4. Mitochondrial Respiration

Oxygen consumption rate (OCR) was quantified using a Seahorse XFe96 Analyzer (Agilent Technologies, Santa Clara, CA, USA), as described previously, with slight modifications [31]. Briefly, cells were plated at 1.10^5^ cells per well in XF96 (Agilent) cell culture plates. OCR measurements were performed in a substrate-free base medium supplemented with 2 mM glutamine (Gibco, Carlsbad, CA, USA) and 10 mM glucose (Sigma-Aldrich). The OCR values are presented as pmoles/min/10^5^ cells. Sequential injections of DPI (20 µM; Sigma) or vehicle DMSO, oligomycin (1 μM; Sigma), DNP (100 µM), and rotenone/antimycin A (0.5 μM; Sigma) were used to determine the main respiratory parameters—in particular, the acute response to DPI, and the impact of the latter on maximal respiration. A modified strategy was used to calculate IC50 values for DPI, rotenone, and antimycin A, as described in Figure 3c. The effects of the concentrations of DPI or standard inhibitors on the OCR variation were compared with the effect of the rotenone–antimycin A mixture, used as a positive control for inhibition of the respiratory chain. This allowed us to define the IC50 of the respiratory activity for each compound.

### 4.5. Apoptosis Assay

Cells were cultured alone or in the presence of DPI (0.4 μM). Three days after drug addition, the cells were harvested and washed with cold PBS, and then resuspended in Annexin-V Binding Buffer (BioLegend, London, UK). Next, the cells were stained with APC-conjugated Annexin-V (BioLegend) and 7-AAD (Sigma-Aldrich) according to BioLegend’s instructions, and then analyzed using a C6 Accuri^®^ flow cytometer and FlowJo^®^ software.

### 4.6. Drug Combination Assay

Cells were seeded in 160 μL of medium at a density of 4 × 10^3^ cells/well, and incubated overnight at 37 °C. They were then exposed to various concentrations of DPI and cytarabine (Ara-C) (Sandoz France Levallois-Perret, France) in a final volume of 200 μL. The proliferation assay was followed, after 72 h of treatment, by a resazurin fluorescence assay. Resazurin (0.1 mg/mL) was added at 20 μL/well and incubated for 4 h at 37 °C in the dark, and then fluorescence was (λ_ex_ = 529.5-19 nm, λ_em_ = 582-36 nm) measured using a CLARIOstar microplate reader. Synergy analysis was performed in the R environment, using the Synergyfinder package [32].

## 5. Conclusions

This work (1) demonstrates that DPI affects AML proliferation in the absence of NOX activity, (2) confirms its inhibitory effect on the MRC, and (3) shows that combining conventional chemotherapy with an MRC inhibitor may help to eradicate the chemotherapy resistance of leukemic cells.

## Figures and Tables

**Table 1:** IC50 values of mitochondrial OCR for various NOX and respiratory chain inhibitors.

Cell line		Mitochondrial OCR IC50					
		KG-1a		THP-1		MV-4-11	
Inhibitors	Target	IC50 (µM)	95% CI (µM)	IC50 (µM)	95% CI (µM)	IC50 (µM)	95% CI (µM)
Rotenone	Complex I	0.55	0.40 to 0.76	1.22	0.83 to 1.80	0.64	0.51 to 0.82
Antimycin A	Complex III	0.26	0.18 to 0.39	0.53	0.40 to 0.69	0.32	0.24 to 0.42
DPI	Flavoproteins	0.2	0.13 to 0.30	1.29	0.91 to 1.82	0.78	0.55 to 1.11

Note: IC50 values (micromolar) were calculated by a nonlinear fit model, and are shown as estimated values with 95% CIs. Abbreviations: IC50, 50% inhibitory concentration; CI, confidence interval.

## Data Availability

Not applicable.

## Supplementary Materials

The following supporting information can be downloaded at: https://www.mdpi.com/article/10.3390/cancers14102485/s1, Figure S1 Kinetic curves of a representative experiment showing the production of DHE and CM-DCFDA in 8 AML cell lines.; Figure S2 Representative apoptosis experiment.
