# Supplementary material for: Diphenyleneiodonium Triggers Cell Death of Acute Myeloid Leukemia Cells by Blocking the Mitochondrial Respiratory Chain, and Synergizes with Cytarabine
Source: Cancers (Basel). 2022 May 18;14(10):2485. doi: 10.3390/cancers14102485 (PMC9140039; doi:10.3390/cancers14102485)
Supplement: Supplementary file 1 [file cancers-14-02485-s001.zip › cancers-1508966-supplementray.pdf]

cancers

MDPI

Supplementary Materials

Diphenyleneiodonium Triggers Cell Death of Acute Myeloid Leukemia Cells by Blocking the Mitochondrial Respiratory Chain, and Synergizes with Cytarabine

Hassan Dakik, Maya El Dor, Jérôme Bourgeais, Farah Kouzi, Olivier Herault, Fabrice Gouilleux, Kazem Zibara and Frédéric Mazurier

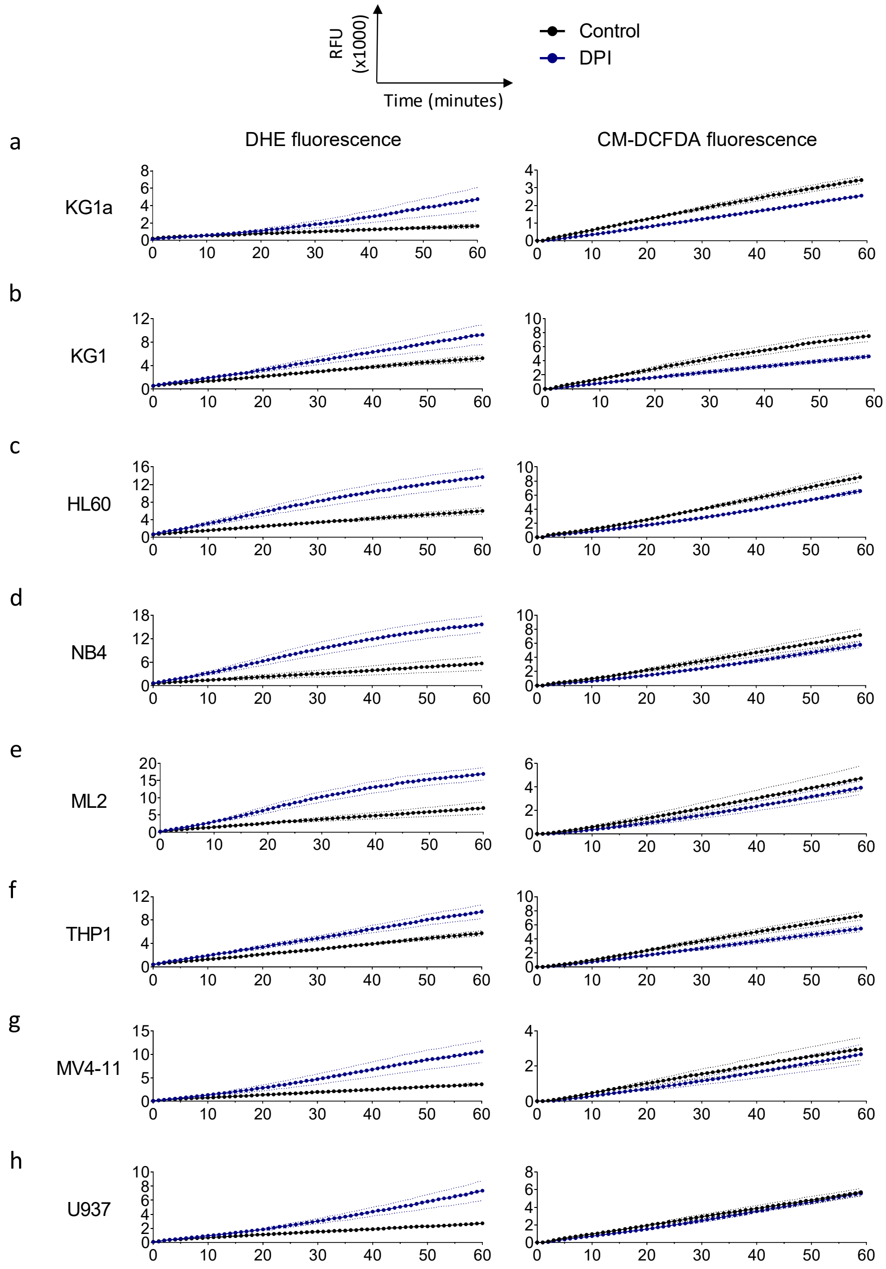

Figure S1. Kinetic curves of a representative experiment showing the production of DHE and CM-DCFDA in 8 AML cell lines.

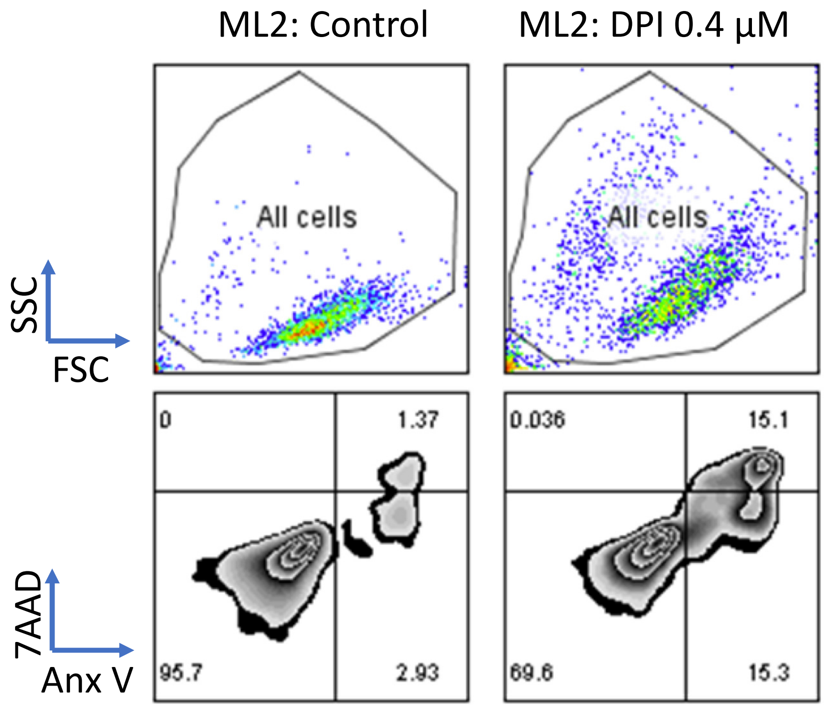

Figure S2. Representative apoptosis experiment.
